# Research Review: Is anxiety associated with negative interpretations of ambiguity in children and adolescents? A systematic review and meta‐analysis

**DOI:** 10.1111/jcpp.12822

**Published:** 2017-10-20

**Authors:** Suzannah Stuijfzand, Cathy Creswell, Andy P. Field, Samantha Pearcey, Helen Dodd

**Affiliations:** ^1^ School of Psychology and Clinical Language Sciences University of Reading Reading UK; ^2^ School of Psychology University of Sussex Brighton UK

**Keywords:** Interpretation bias, anxiety, children, adolescents, development, content specificity

## Abstract

**Background:**

The tendency to interpret ambiguity as threat (negative interpretation) has been implicated in cognitive models of anxiety. A significant body of research has examined the association between anxiety and negative interpretation, and reviews suggest there is a robust positive association in adults. However, evidence with children and adolescents has been inconsistent. This study aimed to provide a systematic quantitative assessment of the association between anxiety and negative interpretation in children and adolescents.

**Method:**

Following systematic searches and screening for eligibility, 345 effects sizes from 77 studies were meta‐analysed.

**Results:**

Overall a medium positive association was found between anxiety and negative interpretation in children and adolescents ($\hat{d}$ = .62). Two variables significantly moderated this effect. Specifically, the association increased in strength with increasing age and when the content of ambiguous scenarios matched the anxiety subtype under investigation.

**Conclusions:**

Results extend findings from adult literature by demonstrating an association in children and adolescents with evidence for content specificity in the association. Age effects imply a role for development. Results raise considerations for when and for whom clinical treatments for anxiety focusing on interpretation bias are appropriate. The vast majority of studies included in the review have used correlational designs and there are a limited number of studies with young children. The results should be considered with these limitations in mind.

## Introduction

Anxiety disorders are among the most prevalent mental disorders, affecting around 6.5% of children and adolescents worldwide (Polanczyk, Salum, Sugaya, Caye, & Rohde, 2015). Anxiety in children has a negative impact on family, educational and social functioning (Settipani & Kendall, 2013; Velting & Albano, 2001) and is associated with future suicidal ideation and depression (c.f. Benjamin, Harrison, Settipani, Brodman, & Kendall, 2013).

In cognitive models (e.g. Kendall, 1985), anxiety is viewed as an emotional, behavioural and cognitive state that is underpinned by threat‐related schemas. These schemas are activated and guide cognitive processing in response to threat or the potential for threat. When an individual has an overactive threat schema, negative cognitive biases result. Cognitive biases can occur at various stages of information processing including attention and interpretation (Muris & Field, 2008). This review focuses specifically on negative interpretation bias, that is, a tendency to interpret ambiguity in a threatening or negative way. This bias has been implicated in cognitive behavioural models of anxiety as having a predisposing (Bar‐Haim, Lamy, Pergamin, Bakermans‐Kranenburg, & van IJzendoorn, 2007; Eysenck, 1992, 1997; Williams, Watts, MacLeod, & Mathews, 1988, 1997), causal (Beck & Clark, 1997), and/or maintaining (Bar‐Haim et al., 2007; Eysenck, 1992, 1997; Mogg & Bradley, 1998; Williams et al., 1988, 1997) role.

To date, three narrative reviews have been conducted that examine the association between anxiety and interpretation bias in children and young people, covering literature up to 2008 (Blanchette & Richards, 2009; Castillo & Leandro, 2010; Muris & Field, 2008). Taken together these reviews tentatively conclude that anxious children and adolescents are likely to show a negative interpretation bias. They also highlight the inconsistency in findings across studies and several unanswered questions. No previous reviews have included a meta‐analysis or claimed to be systematic and none directly tackle the issue of moderators. Thus, the aim of the present paper is to conduct a systematic review and meta‐analysis of the association between anxiety and negative interpretation bias in children and adolescents, taking into account a range of potential moderators.

Studies vary on a range of factors that may moderate the association between negative interpretation and anxiety in children and adolescents. These factors can be grouped together as ‘Population’ factors, those that relate to the participants in the study (e.g. age, whether the focus of the study was a clinical or nonclinical sample); and ‘Procedural’ factors, those that relate to the way in which the study was designed and conducted (e.g. which task was used to assess interpretation bias and who the informant for the anxiety measure was). Careful consideration of moderators is important as it may explain some of the inconsistencies apparent in the literature and provide important insights for treatment. The following sections briefly outline the relevant population and procedural variables that will be assessed as moderators in this review.

## Population variables

### Population focus

Studies vary in whether they focus on community or clinical populations. Here, we include all studies examining the association between anxiety and interpretation bias, including those that focus on clinical samples and those that focus on community samples. Larger effect sizes may be expected in studies using a clinical versus control design than a high versus low community sample design given that the difference in anxiety levels between groups will typically be greater in the former. For the same reason, a larger effect size would be expected when a clinical group are compared to a screened ‘nonanxious’ control group as opposed to an unscreened community sample or a different clinical population (Bar‐Haim et al., 2007).

### Anxiety subtype

Studies with both community and clinical samples vary in terms of whether they look at general anxiety or a specific subtype of anxiety (e.g. social anxiety or total anxiety score). If negative interpretation bias is a feature of a specific type of anxiety then effect sizes will be stronger in some studies than others, dependent on the anxiety subtype considered.

There are also inconsistent results depending on whether the focus is on trait/state anxiety. Although only a few studies have examined state anxiety, there is evidence that both trait and state anxiety may be associated with a negative interpretation bias. However, findings are inconsistent (e.g. Muris, Rapee, Meesters, Schouten, & Geers, 2003; Salemink & Wiers, 2012).

### Demographics

Participant age and sex also vary widely across studies, but the effect of these sample characteristics is unclear. Age is sometimes considered as a covariate or moderator in studies examining interpretation bias and anxiety with mixed results (e.g. Blossom et al., 2013; Waite, Codd, & Creswell, 2015; Waters, Zimmer‐Gembeck, & Farrell, 2012). To our knowledge, no study has assessed sex within the context of this association.

### Comorbidity

Clinical studies vary in whether and how they deal with comorbid disorders; participants with comorbid diagnoses may be included, excluded or comorbidity may not be assessed. As negative interpretation bias has also been found in other common comorbid psychiatric disorders such as depression and externalising disorders (Mathews & MacLeod, 2005; Reid, Salmon, & Lovibond, 2006) (although see. for example, Epkins, 1996; Leung & Wong, 1998), inclusion of those with comorbid disorders may result in the association between negative interpretation bias and anxiety appearing stronger than it would in a ‘pure’ anxious group.

## Procedural variables

### Type of task

Research assessing interpretation bias in children and young people typically uses one of two task formats. Ambiguous scenario tasks (Barrett, Rapee, Dadds, & Ryan, 1996) are the most commonly used. Here, participants are presented with ambiguous social and nonsocial vignettes (via written, auditory, pictorial or a combination, stimuli) and asked to either choose an ending for each vignette from a list or to generate their own. An alternative task is based on lexical knowledge. For example, homophones and/or homographs that have a threat and nonthreat interpretations such as berry/bury and sink (kitchen)/sink (boat) (i.e. Gifford, Reynolds, Bell, & Wilson, 2008) might be used. Typically in this type of task, interpretation is evaluated by asking participants to select an image that matches the word they heard or to use the word in a sentence. Even within the same study inconsistent results have been found between these different tasks (e.g. Waters, Wharton, Zimmer‐Gembeck, & Craske, 2008). The extent to which the nature of the task influences the association between anxiety and interpretation bias in children and young people remains unclear.

### Response formats

Both ambiguous scenario tasks and lexical tasks designed to measure interpretation bias may use open or forced choice response formats, or create a composite of the two. These response formats require active and passive information generation, respectively, which may influence responses and subsequent conclusions regarding bias (Ozuru, Best, Bell, Witherspoon, & McNamara, 2007; Ozuru, Briner, Kurby, & McNamara, 2013).

### Dependent variable

Studies also vary in the dependent variable used to capture interpretation bias. For example, in an ambiguous scenarios task: threat interpretation, threat frequency, threat threshold or a composite of all three may be used (e.g. Muris, Merckelbach, & Damsma, 2000). It is possible that some measures better capture anxiety‐related interpretation biases than others, which could explain some variance in effect sizes reported across the literature.

### Type of scenario

The ambiguous scenarios task also varies by the type of scenario assessed (e.g. social, nonsocial, physical or a response to a range of scenarios to create ‘general scenarios’). This is not typically true of lexical tasks as they are limited by the words available in the English language that possess the required properties (homograph/homophone).

### Content specificity

According to the content specificity hypothesis, (Beck,^1976^), the relationship between interpretation bias and anxiety is expected to be stronger when the interpretation content matches the anxiety subtype. The majority of studies examining interpretation bias and anxiety in young people do not examine content specificity. However, as outlined above, some ambiguous scenario tasks use specific types of scenario that align with specific subtypes of anxiety (e.g. social scenarios/social anxiety). To date there has been no systematic review of whether the bias–anxiety association is stronger when there is a content match than when there is not.

### Anxiety measure informant

The individual providing information about the young person's anxiety also varies across studies: it may be a teacher, parent or the child/adolescent participant. This may affect the strength of the association between bias and anxiety, particularly given that studies differ on whether the same or different informants report on bias and anxiety.

## Aims and scope

The overall aim of the present study is to provide a systematic quantitative assessment of the relationship between negative interpretation and anxiety in children and adolescents, and to evaluate potential moderators of this relationship. The review takes a broad scope with regard to anxiety and includes research that focuses on clinical anxiety as well as research focused on normal individual variation in anxiety levels, both trait and state. Data were drawn from studies with a range of methods including, but not limited to experimental, cross‐sectional, and longitudinal designs that adhered to our eligibility criteria.

## Methods

### Eligibility criteria

Hierarchical eligibility criteria to screen abstracts and full texts for inclusion were:

1. *The paper must be available in English*.
2. *The paper must be an original study, not a review*.
3. *The paper must investigate a human child, adolescent or youth population*. Papers were accepted that reported a maximum age = 21 years and mean age <18 years.
4. *Primary focus must be on typically developing children*. Children with atypical development were excluded as these children may have particular propensities to anxiety and/or may have particular patterns of information processing that could influence their interpretation of ambiguity.
5. *A sound and standardised measure of anxiety or fear should be used for all participants, including clinical and subclinical samples*. The review includes studies focused on clinical and subclinical anxiety and fear, as well as specific subtypes of anxiety (e.g. social anxiety, separation anxiety, generalised anxiety). To be included papers must have utilised a sound and standardised measure of the type of anxiety in question, that is, interviews must be semistructured or structured, and completed by child/adolescent, parent or both. Anxiety questionnaires could have been completed by either child, parent or teacher, but had to show internal consistency of at least 0.7 and evidence of construct validity. Finally, the age range of participants must be appropriate (±1 year of the suggested age range) for the measure used.
6. Papers were included where a correlational or between‐groups design was used. Where participants were prescreened into high‐ and low‐anxiety groups: *Papers should determine high anxiety by either: (a) A clinical diagnosis via a standardised diagnostic interview; (b) All participants in high‐anxiety group must score more than 1 SD above a normative mean on a standardised measure of anxiety or fear; (c) All participants in the high‐anxiety group must score above a cut‐off recommended by the authors of the measure used (sensitivity analysis must have been conducted to validate this cut‐off). No differences in age and sex should have been found between the high‐anxiety group and the corresponding comparison group. Where these criteria were not met, papers were included only if a continuous measure of anxiety could be obtained to produce a correlation*.
7. *The sample should not represent a restricted range of anxiety*. Those including only clinical/high anxious or at risk samples were excluded.
8. *Papers using cognitive bias modification are eligible only if a pre‐modification measure of the relationship between interpretation bias and anxiety was reported,* and, if so, this effect size was extracted for this the meta‐analysis.
9. *The measure of interpretation bias captured the extent to which participants interpreted ambiguity as threatening or negative and/or the child/adolescents readiness to perceive threat, that is, Reduced Evidence for Danger (RED)*.^1^
10. *Where open‐ended interpretations of ambiguous scenarios were coded, inter‐rater reliability must be at least 0.7 for inclusion,* unless open‐ended responses were significantly associated with forced choice answers.
11. *Full‐text access must be available to be able to code and extract all the information necessary for the meta‐analysis*.
12. *Appropriate statistics regarding the relationship between interpretation bias and anxiety should be available*. If these were not immediately accessible from the paper authors were contacted.

### Information sources

Studies were identified through searches on the databases: PubMed, Psych Info/Psych Articles, Web of Science, Google Scholar, NHS Evidence database. The searches were conducted on all papers from 1990, when the first studies examining interpretation bias and child anxiety were published, to the present day. A check for papers prior to 1990 was conducted and no papers conforming to the age limit were identified. Searches were conducted on 6 August 2015. Additionally, the references of previous reviews (i.e. Blanchette & Richards, 2009; Castillo & Leandro, 2010; Muris & Field, 2008) and all accepted papers were checked for relevant papers. Finally, first authors and corresponding authors of accepted papers were contacted to request any relevant unpublished work.

### Search

Two sets of search terms were used. One set of terms focused on interpretation bias and anxiety, including anxiety subtypes, while the second set specifically identified papers using cognitive bias modification (CBM) that may have been missed by the first search terms. The exact search terms used can be found in Appendix S2.

### Study selection

Study selection procedures adhered to PRISMA guidelines (Liberati et al., 2009). To select studies, abstracts from all sources were first screened against the eligibility criteria, followed by full texts. A paper could be excluded at any stage of the screening process on the basis of a ‘no’ response to any of the eligibility criteria; the first criterion that was not met was recorded as the reason for rejection. Where criteria were coded as unclear (in the absence of any ‘no’ codes) at the abstract stage, papers went through to full text screening. Where particular criteria were not applicable they were not coded. Those papers that were accepted via the full paper screening were then coded according to the coding criteria (see below and Table A1 in Appendix S1), and appropriate data were extracted. Duplicates were removed at both the abstract and full paper screening stages. Full or partial overlap of data between published and unpublished data was checked for during this process and unpublished data excluded as a duplicate.

### Data collection process

A postgraduate student piloted the eligibility criteria and search terms and eligibility criteria were altered accordingly (specifically the word ‘human’ was added to criteria 3 regarding age of participants to ensure only papers on human populations were accepted). After completion of the piloting two coders (both postgraduate students) checked the first 208 abstracts against the eligibility criteria. On the basis of these 208 abstracts a high level of inter‐rater reliability between coders was found for reject/accept decisions (*κ *= .91, *p *<* *.001). The remaining abstracts were coded by the first coder.

To ensure reliability of the criteria for full paper screening, the same two coders both assessed 20 full texts against the eligibility criteria. Agreement between the two coders was found on 90% of the papers.^2^ Any disagreements between coders at either stage of the screening were discussed with the first author to reach a consensus. The first coder then coded the remaining full texts. Once all the full texts had been screened, the first author then extracted the relevant statistics (effect sizes; sample sizes; means and standard deviations where effect sizes were not available; and demographic information including, mean age, and percentage of males in the sample) from the accepted full texts.

### Data items

Papers were coded for a range of sample characteristics and moderator variables. A detailed description of coding criteria for each characteristic and level of all moderators is provided in Table A1 in Appendix S1. Where papers had investigated potential mediators or moderators of the association between negative interpretation and anxiety, the moderator/mediator of interest was coded along with the resultant associations with anxiety and negative interpretation separately.

### Risk of bias within individual studies

Attempts were made to reduce risk of bias within the studies included in the meta‐analysis in two ways. Firstly, studies were only included if they adhered to our strict eligibility criteria regarding methods. Secondly, characteristics related to quality such as control group, measures used and whether the study was published or unpublished were included as moderators within the analysis to investigate whether these affected results.

### Summary measures

Cohen's *d* was extracted for all papers included in the meta‐analysis. Where Cohen's *d* was not available for the association of interest, means and standard deviations were used to compute *d*. If these were also not available, *t*‐statistics and degrees of freedom were used. Where studies reported a correlation *r*, this was converted to Cohen's *d* using the formula described by Rosenthal (1994) on p.239. Effect sizes were coded in the same direction so that a positive *d* always indicated that those with higher anxiety showed greater negative interpretation. Where correlations were included, positive correlation coefficients always indicated that as anxiety/fear scores increased so did negative interpretation scores prior to transformation to *d*.

### Planned method of analysis

Most studies yielded more than one effect size due to multiple outcome measures being used or the same outcome being taken at multiple time points. To account for the dependency this created among effect sizes within studies a multilevel approach was used, in which effect sizes (level 1) were nested within studies (level 2). Effect sizes were allowed to vary across studies as a random effect, and moderators were treated as fixed effects. The model fitted is described by:

$$d_{j}=\gamma_{0}+\gamma_{1}Z_{1j}+\gamma_{2}Z_{2j}+\cdots \gamma_{p}Z_{pj}+\mu_{j}+e_{j}$$

Which states that effect size, *d*, in study *j* are predicted from the mean effect size across studies, γ_0_, study characteristics, *Z*_1_… *Z*_*p*_, and their associated parameter estimates, γ_1_… γ_*p*_. The deviation of the effect in study *j* from the overall mean is reflected in the residual, μ_*j*_, which is assumed to have a normal distribution with variance σ_μ_. The sampling error for study *j* is reflected in *e*_*j*_, which has a normal distribution with variance σ_*j*_. When no moderators are included, this model reduces to:

$$d_{j}=\gamma_{0}+\mu_{j}+e_{j}$$

Which states that the effect, *d*, in study *j*, is equal to the mean effect across studies γ_0_, its deviation from that mean, μ_*j*_, and the sampling error for that study, *e*_*j*_.

The models were fitted using R 3.2.4 (R Core Team, 2015) using the rma.mv()function in the metafor package (Viechtbauer, 2010), data processing was conducting using the reshape (Wickham, 2007) and car packages (Fox & Weisberg, 2011), and sensitivity analysis was conducted with the weight package (Coburn & Vevea, 2016). To be included as a level within a moderator analysis, at least two effect sizes had to be available.

Funnel and forest plots of effect sizes aggregated within studies (so that each study was represented by one effect size) were used to assess outliers, as well as Cooks distance where influence was assessed by checking whether dfbetas were greater than one (Viechtbauer & Cheung, 2010).

### Risk of bias across studies

In addition to reducing publication bias by requesting and including unpublished work, publication bias was also assessed using a funnel plot with statistical tests of asymmetry.

### Additional analysis

Sensitivity analyses were conducted using the trim and fill method (Duval & Tweedie, 2000a, 2000b) and a priori weight functions (Vevea & Woods, 2005).

## Results

### Study selection

**Figure 1:**
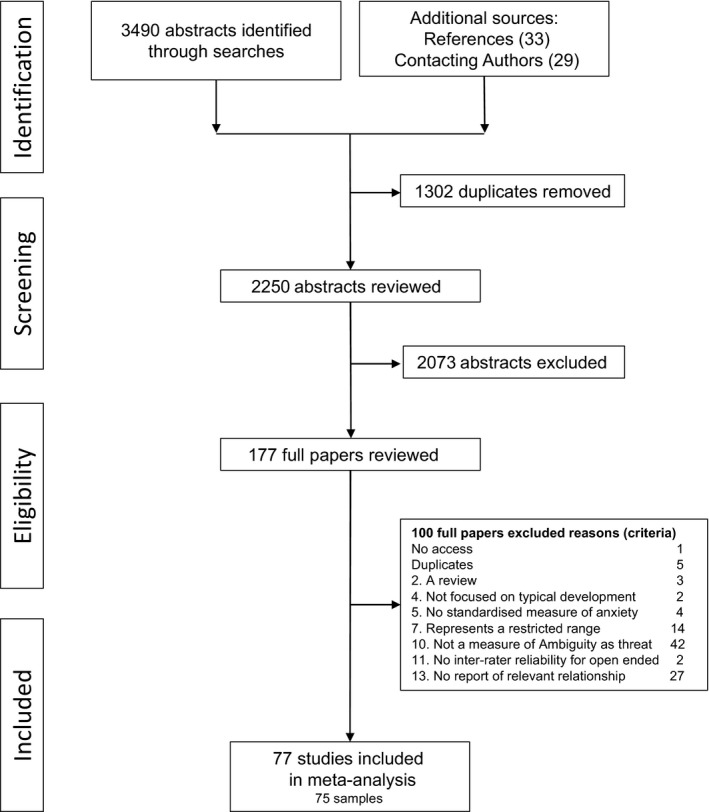
Flow chart of abstracts and papers accepted through the eligibility screening process

Six authors were contacted as the information required to calculate an effect size was not available in the paper. Three authors were able to provide the necessary information and the studies were therefore included. After the complete selection process, a total of 77 studies representing 75 samples were included in the meta‐analysis, resulting in the inclusion of 345 effect sizes (see Figure 1 for flow chart of numbers screened and accepted at each stage of the selection procedure).

### Study characteristics and results from individual studies

**Figure 2:**
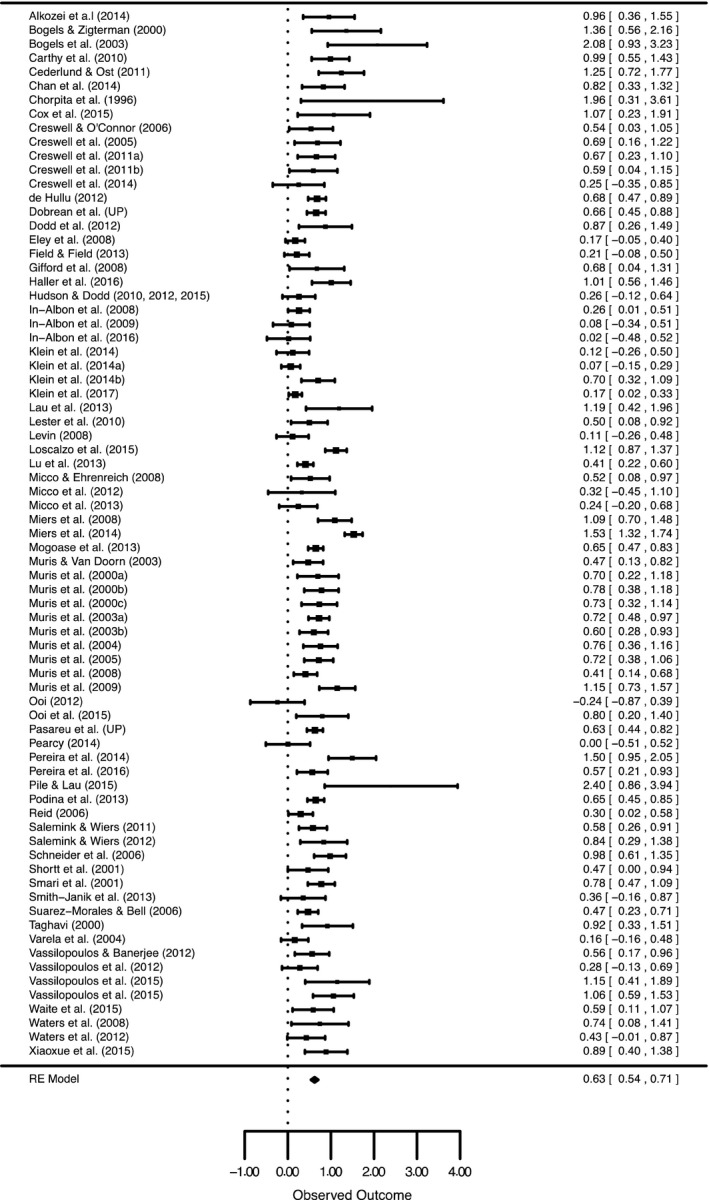
Forest Plot of all Studies included in the Meta‐analysis and Moderation Analyses. Values represent the mean effect sizes within a study

Following assessments, no studies yielded an effect size that was an outlier. Therefore, the total sample included 11,507 children and adolescents with an average age across studies of 11.19 years old (*SD *= 1.28, min = 2, max = 22). Eighteen studies (16 samples) focused on anxiety and interpretation bias within clinical samples and 57 studies focused on anxiety and interpretation bias within community samples. Table 1 lists all studies included within the meta‐analysis and their characteristics. Aggregated effect sizes within each study, along with their confidence intervals, can be seen in Figure 2. Note that the statistics in the following sections are from a multilevel model that factors in the dependency between effect sizes from the same study, whereas the overall effect size in Figure 2 is based on a model in which effect sizes within studies are aggregated so that each study contributes only one effect size.

**Table 1:** Study characteristics related to each sample included in the between‐groups analysis

Sample no.	Study label	*N*	Mean age (*SD*)	*n* (Clinical)	*n* (Community)	*d*	No. ES
1	Alkozei et al. (2014)	50	10.07 (1.91)	50	25	0.96	18
2	Cederlund & Ost (2011)	75	11.50 (1.80)	38	38	1.25	4
3	Creswell et al. (2011a)	94	–	–	92	0.67	1
4	Bögels et al. (2003)	25	12.20 (2.90)	6	25	2.08	1
5	Bögels & Zigterman (2000)	30	12.45 (3.00)	30	16	1.36	6
6	Carthy et al. (2010)	88	–	46	42	0.99	2
7	Creswell et al. (2011b)	56	–	–	65	0.59	1
8	Creswell and O'Connor (2006)	65	–	–	65	0.54	1
9	Creswell et al. (2005)	60	10.61 (2.36)	27	33	0.69	1
10	Creswell et al. (2014)	52	9.66 (1.02)	80	40	0.25	2
11	Hudson & Dodd (2010, 2012, 2015)	117	8.73 (0.37)	36	81	0.26	3
12	Eley et al. (2008)	300	–	–	300	0.17	3
13	Field & Field (2013)	187	10.07 (0.88)	–	187	0.21	27
14	Gifford et al. (2008)	43	9.98 (1.19)	32	23	0.68	2
15	In‐Albon et al. (2009)	96	8.94 (2.20)	102	42	0.08	6
16	In‐Albon, Klein, Rinck, Becker, and Schneider (2008)	252	9.69 (1.80)	–	265	0.26	6
17	Klein et al. (2014)	108	10.10 (1.40)	–	108	0.12	24
18	Lau et al. (2013)	36	9.33 (1.33)	–	36	1.19	7
19	Lu et al. (2013)	459	10.98 (0.90)	–	459	0.41	4
20	Mogoase et al. (2013)	571	13.01 (1.19)	–	571	0.65	2
21	Muris et al. (2009)	120	10.86 (1.07)	–	120	1.15	3
22	Podina et al. (2013)	423	11.69 (3.63)	–	423	0.65	1
23	Salemink and Wiers (2012)	64	14.50 (0.60)	–	65	0.84	2
24	Smith‐Janik et al. (2013)	59	9.59 (0.83)	–	59	0.36	12
25	Waters et al. (2012)	85	10.43 (1.41)	–	85	0.43	6
26	Lester et al. (2010)	92	9.13 (1.41)	–	92	0.5	3
27	Micco & Ehrenreich (2008)	80	10.96 (2.12)	40	40	0.53	2
28	Micco, Hirshfeld‐Becker, Henin, and Ehrenreich‐May (2013)	80	–	40	40	0.24	9
29	Miers et al. (2008)	209	13.68 (0.98)	–	73	1.09	1
30	Muris et al. (2007)	216	10.88 (0.95)	–	216	0.41	3
31	Vassilopoulos et al. (2012)	94	10.50 (0.50)	–	94	0.28	1
32	Levin (2008)	111	14.70 (–)	–	111	0.11	2
33	Muris et al. (2004)	113	10.10 (1.00)	–	113	0.76	1
34	Muris et al. (2000a)	76	10.40 (1.20)	–	76	0.7	1
35	Muris et al. (2003a)	299	9.80 (1.20)	–	299	0.72	4
36	Muris et al. (2003b)	156	10.70 (0.90)	–	156	0.60	10
37	Muris et al. (2000b)	252	10.10 (1.30)	28	224	0.78	2
38	Muris et al. (2000c)	105	10.20 (1.20)	–	105	0.73	4
39	Ooi (2012)	40	4.71 (0.86)	–	44	−0.24	1
40	Pereira et al. (2014)	80	8.84 (1.23)	–	80	1.5	1
41	Reid (2006)	192	–	–	192	0.3	4
42	Salemink & Wiers (2011)	158	14.50 (0.50)	–	158	0.59	6
43	Schneider et al. (2006)	143	11.57 (1.68)	–	143	0.98	3
44	Smari et al. (2001)	184	–	–	184	0.78	60
45	Shortt et al. (2001)	124	8.93 (2.12)	113	–	0.47	9
46	Suarez‐Morales & Bell (2006)	292	10.46 (0.55)	–	292	0.47	9
47	Taghavi (2000)	57	13.39 (2.33)	17	40	0.92	3
48	Waters et al. (2008)	39	9.95 (1.25)	19	19	0.75	6
49	Vassilopoulos and Banerjee (2012)	110	11.50 (0.50)	–	210	0.57	1
50	Muris & Van Doorn (2003)	138	10.50 (1.20)	–	138	0.47	1
51	Chorpita et al. (1996)	12	11.30 (1.78)	4	8	1.96	1
52	Muris et al. (2005)	157	10.80 (0.95)	–	157	0.72	1
53	Varela et al. (2004)	154	11.46 (1.10)	–	154	0.16	1
54	Vassilopoulos et al. (2015a)	38	10.40 (0.30)	–	38	1.15	3
55	Vassilopoulos et al. (2015b)	89	11.20 (0.60)	–	89	1.06	1
56	In‐Albon et al. (2016)	70	10.21 (1.55)	35	28	0.02	11
57	Cox et al. (2015)	29	11.43 (0.28)	–	29	1.07	1
58	Fu et al. (2015)	73	14.15 (1.60)	–	73	0.89	1
59	Haller et al. (2016)	95	16.67 (1.05)	–	95	1.01	2
60	Pile & Lau (2015)	17	16.53 (0.62)	–	17	2.4	2
61	Pereira et al. (2016)	131	9.70 (1.50)	131	–	0.57	4
62	Păsăreu et al. (2015)	480	13.19 (1.67)	–	480	0.63	2
63	Dodd (2012)	50	16.68 (1.02)	–	50	0.87	2
64	Dobrean et al. (2015)	366	12.90 (1.86)	–	366	0.66	3
65	Waite et al. (2015)	80	12.24 (0.99)	40	40	0.59	1
66	Micco et al. (2012)	27	5.26 (1.14)	–	27	0.325	1
67	Miers et al. (2014)	559	13.90 (1.63)	–	559	1.53	3
68	Ooi et al. (2015)	50	4.00 (0.50)	–	50	0.8	2
69	Chan et al. (2015)	75	16.64 (0.67)	–	74	0.825	1
70	Hullu (2012)	389	13.56 (0.69)	–	284	0.68	1
71	Pearcey (2014)	72	8.62 (1.05)	42	31	0.003	1
72	Loscalzo et al. (2015)	329	15.36 (1.12)	25	204	1.12	3
73	Klein et al. (2014a)	333	9.95 (1.25)	–	381	0.07	2
74	Klein et al. (2014b)	125	9.24 (1.65)	103	21	0.70	1
75	Klein et al. (2017)	678	14.37 (1.16)	–	678	0.174	2

N‐Dashes (–) indicate the data were not available for extraction. Multiple effect sizes were taken from each study therefore values in the table represent aggregated sample size per association, aggregated mean age, aggregated standard deviation of age, aggregated sample size from a clinical population and aggregated sample size from a community population, number of effect sizes taken per study and aggregated effect size. Because numbers are aggregated within studies, the total aggregated sample size may appear different to the sum of the aggregated clinical and community samples respectively. No. ES = number of effect sizes drawn from each sample.

### Synthesis of results

The overall meta‐analysis yielded a population estimate of the association between anxiety and negative interpretation in children and adolescents of $\hat{d}$ = .62 of a standard deviation (Table 2). There was significant variation between effect sizes, *Q *=* *1,452.28, *p *<* *.001.

**Table 2:** Meta‐analytic results

	*N studies*	*k*	$\hat{d}$	95% CI	*Q* a	*p*
Overall						
Anxious versus Nonanxious	75	345	.62**	0.53, 0.70		
**Moderators**						
Population‐related moderators						
Population Focus	75	317			0.35	.555
Clinical	18	110	.58***	0.41, 0.76		
Community	57	207	.64***	0.54, 0.73		
Control group	17	134			2.77	.734
Screened nonanxious	9	52	.51***	0.28, 0.73		
Diagnosed nonanxious	9	43	.70***	0.46, 0.95		
Not social anxiety	4	21	.58	−0.06, 1.24		
Not separation anxiety^c^	1	2	−.22	−0.56, 0.11		
Clinical externalising^c^	3	4	.57	−0.77, 1.91		
Correlation^c^	3	12	.46*	0.05, 0.86		
High trait anxiety^d^	1	1	–	–	–	–
Low trait anxiety^d^	1	1	–	–	–	–
Comorbidity with Other Anxiety Disorder	18	82			0.59	.441
Included	16	77	.61***	0.43, 0.79		
Excluded^c^	3	5	.69***	0.03, 1.35		
Comorbidity with depression	–	–			–	–
Included	12	62	.66***	0.45, 0.86		
Exclude	1	1	–	–		
Comorbidity with another disorder^b^	15	63			.01	.939
Included	8	41	.65***	0.31, 0.98		
Excluded	7	22	.60***	0.37, 0.83		
Anxiety subtype	75	317			9.92	.193
General anxiety	55	201	.61***	0.53, 0.69		
OCD^c^	3	3	.55**	0.21, 0.89		
Phobias	5	10	.43**	0.18, 0.69		
Separation anxiety	9	15	.36***	0.17, 0.55		
Social anxiety	27	57	.72***	0.51, 0.92		
State anxiety^c^	4	6	.62***	0.50, 0.74		
Other anxiety	4	19	.41	−0.03, 0.84		
PTSD	1	1	–	–	–	–
Procedural moderators						
Task type	75	318			1.18	.277
Ambiguous scenarios	72	310	.63***	0.54, 0.71		
Lexical tasks^c^	5	8	.54**	0.11, 0.96		
Response format	75	318			5.78	.056
Forced choice	57	209	.66***	0.56, 0.76		
Open	31	84	.51***	0.39, 0.63		
Open and forced choice	5	25	.36***	0.23, 0.48		
Dependent variable	75	317			2.87	.237
Threat interpretation	75	241	.68***	0.58, 0.78		
Threat frequency	10	39	.78***	0.66, 0.90		
Threat threshold	9	37	.68***	0.58, 0.78		
Scenario type	72	268			1.42	.841
Social	18	46	.60***	0.44, 0.78		
General	60	173	.62***	0.52, 0.72		
Separation	7	29	.49***	0.26, 0.72		
Phobias^c^	3	5	.29	−0.06, 0.65		
Physical information	6	15	.51**	0.19, 0.82		
Match: Scenario and anxiety subtype	75	318			4.24	.039
No match	70	289	.59***	0.50, 0.68		
Match	13	29	.79***	0.53, 1.05		
Informant measure anxiety	74	317			2.77	.250
Child	56	215	.65***	0.56, 0.75		
Parent	7	21	.50***	0.19, 0.80		
Child and parent	17	81	.54***	0.36, 0.72		
Teacher^d^	0	0	–	–		

The first level under each moderator is the reference category.

^a^ *Q* for comparisons of the subtypes of the moderator.

^b^Other disorder refers to an externalising or other psychiatric disorder rather than an internalising disorder.

^c^Moderator levels identified with small numbers of studies and effect sizes that may influence the generalisability of the findings.

^d^One or no effect sizes were available for these moderator levels therefore the level was not included in the moderation analysis.

*p =* *.05; **p =* *.01; ***p < .001.

Table 2 shows all moderation analyses, and separate meta‐analyses for each level of the moderator, as well as their respective confidence intervals (see Appendix S1 for a list of all moderators and their definitions). The first level listed under the title of each moderator indicates the reference group used in the moderation analyses.

### Moderation by population variables

As shown in Table 2, variation among effect sizes was not accounted for by population focus. Therefore, the effect sizes from clinical and community samples were combined for all remaining moderation analyses.

There were not enough effect sizes (*k* ≤ 1 for all levels except social anxiety and separation anxiety) available to conduct an analysis across clinical anxiety disorders. As there were enough effect sizes comparing social anxiety (*k* = 21) and separation anxiety (*k* = 2) and other anxiety disorders we included two additional levels in the planned overall control group analysis: ‘Not social anxiety’ and ‘Not separation anxiety’ respectively (see Table 2; for descriptions of these levels see Table A1 in Appendix S1). Variation was not found to be accounted for by control group. Given these results, the associations with ‘Not social anxiety’, the ‘Not separation anxiety’ or the ‘Clinical Externalising’ control groups were excluded from the remaining analyses to allow a clear picture of the association between negative interpretation and anxiety in children and adolescents (for descriptions of these levels see Table A1 in Appendix S1).

**Figure 3:**
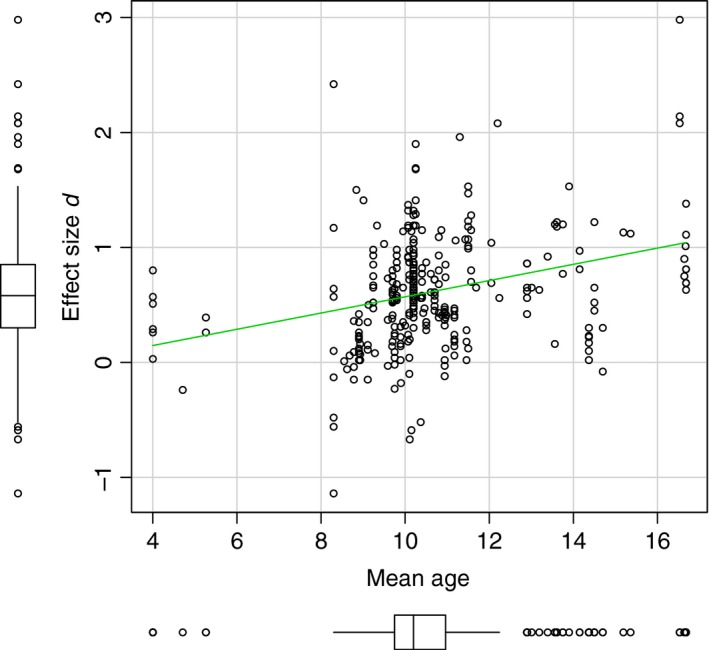
Scatterplot with box plots to show the relationship between the mean age (in years) and corresponding effect size (*d*) from each study included in the meta‐analysis. The green line represents a parametric regression line

Variation among effect sizes was not significantly accounted for by the inclusion/exclusion of comorbidity with another anxiety disorder or comorbidity with another psychiatric disorder (Table 2). Furthermore, variation among effect sizes was not accounted for by anxiety subtype, (descriptors of all moderators and their respective levels can be found in Table A1 in Appendix S1). Nor was variance in effect sizes accounted for by sex, *b *= −.0003 [−.009, .009], *p *=* *.940. In contrast, age significantly predicted effect size magnitude, *b *=* *.06 [03, .10], *p *<* *.001; with increasing age, the association between negative interpretation and anxiety in children and adolescents also increases. To provide greater insight into the significant moderation by age, mean age per study was plotted against the study's corresponding aggregated effect size (see Figure 3).

### Moderation by procedural variables

As Table 2 indicates, variation among effect sizes was not significantly accounted for by the task used to assess interpretation of ambiguity, open versus forced choice responses, scenario type, the dependent variable assessed or anxiety measure informant. However, content specificity was a significant moderator (see Table 2); when the scenario content matched the anxiety subtype, the association between negative interpretation and anxiety was larger than when they did not match.

### Risk of bias within studies

Whether the study was unpublished (15 samples, 64 effect sizes) or published (61 samples, 254 effect sizes) did not significantly account for variation among effect sizes *Q *=* *2.99, *p *=* *.0838. Individual meta‐analyses indicate that a robust effect size was present among both published ($\hat{d}$ = .63 [.55, .72], *p *<* *.001) and unpublished ($\hat{d}$ = .54 [.27, .80], *p *<* *.001) work.

### Risk of bias across studies

To reduce publication bias through the inclusion of unpublished works, 58 researchers were contacted (four could not be contacted) and 70% responded to our email request. Of these, 21 authors provided additional unpublished manuscripts or data resulting in 29 further studies assessed for eligibility, 24 were accepted (since this request 10 of these papers have been either published or are under review at the time of writing, as reflected in the references in Appendix S3).

**Figure 4:**
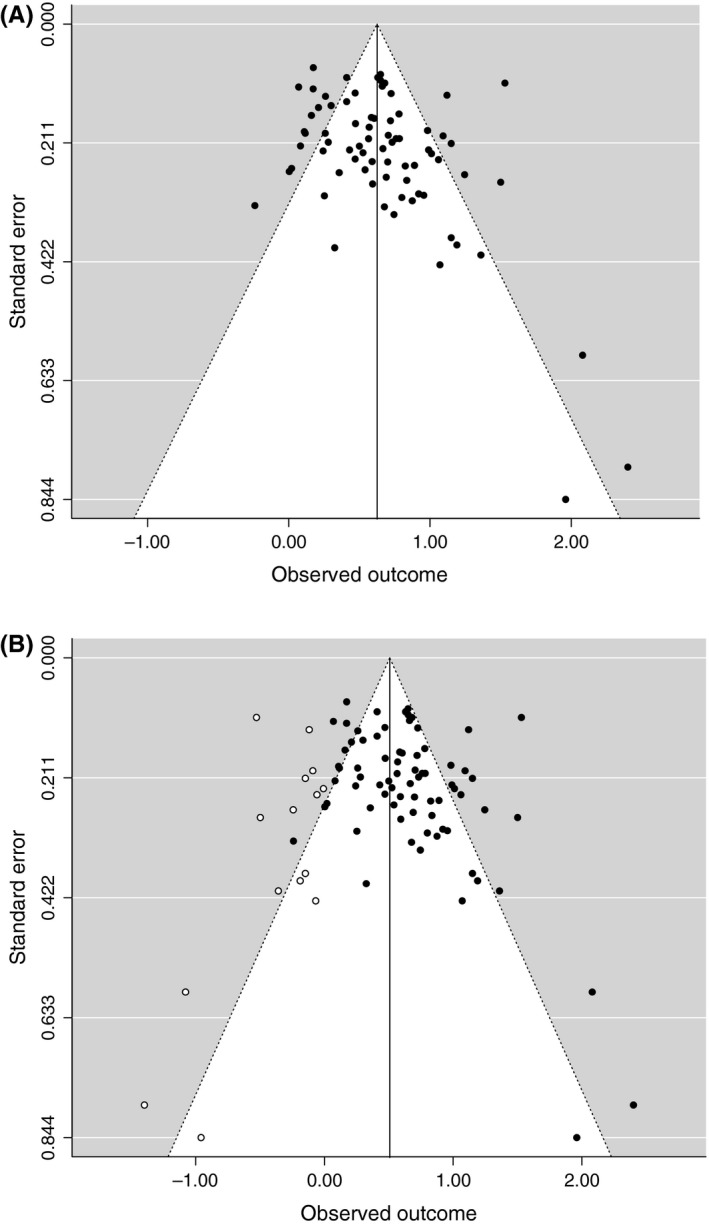
(A) Funnel plot for publication bias: Cohen's *d* to standard error. (B) funnel plot with trim and fill sensitivity test

The funnel plot to assess publication bias was found to be asymmetrical (τ* *= .21, *p *=* *.0072; *z *=* *3.30, *p *<* *.001; see Figure 4A), this appears to be mainly driven by three studies with large effect sizes but small sample sizes. Studies with small samples and negative effects were absent, hence the asymmetry.

### Additional analysis

The sensitivity method, trim and fill, indicated that 15 more studies would be required to satisfy symmetry (see Figure 4B). If these extra studies were entered with a *d* of 0, the association between negative interpretation and anxiety would be only slightly smaller ($\hat{d}$ = .51, *p* < .001). Following Vevea and Woods (2005), a prespecified sensitivity analysis was conducted using a priori weight functions. The estimate from the overall meta‐analysis proved to be quite robust, suggesting publication bias is unlikely to be an important influence on the results (adjusted model estimates ranged from $\hat{d}$ = .51−.61).

## Discussion

### Summary of evidence

Our meta‐analysis indicated that there is a medium‐sized overall association between negative interpretation and anxiety in children and adolescents, and that this effect is robust across clinical and community samples as well as across comparison groups for clinical samples. There was significant heterogeneity across studies, which were partially accounted for by child/adolescent age and whether the content of the interpretation‐task matched the specific subtype being assessed.

The overall findings are consistent with adult reviews on the association between interpretation bias and anxiety (Blanchette & Richards, 2009; Mobini, Reynolds, & MacKintosh, 2013) and the previous narrative reviews of the child and adolescent literature (Muris & Field, 2008). There is no equivalent meta‐analysis assessing the association between negative interpretation and anxiety in adults, therefore as yet the effect sizes cannot be compared. However, to give some context, the population effect size estimate of $\hat{d}$ = .62 is larger than that found between anxiety and attention bias in children and adolescents ($\hat{d}$ = .21; Dudeney, Sharpe, & Hunt, 2015).

### Evidence for an age effect

The results indicated that as age increases the association between negative interpretation and anxiety increases in strength. Dudeney et al. (2015) also found age effects in their meta‐analysis of attention bias and anxiety in children and adolescents. Taken together, these findings indicate that age/development may moderate the association between anxiety and cognitive biases more broadly.

The analysis presented in Figure 3 indicates a positive linear relationship between the magnitude of the effect size and age, however, it is important to note that the vast majority of studies included had a participant mean age above 8 years. There are very few effect sizes available for children below 8 years old (*N studies *= 4, *k *=* *9), with none available for children between 6 and 8 years old. This limits the conclusions that can be drawn about interpretation bias and anxiety in young children which is a noteworthy omission as anxiety symptoms cause significant impairments in children as young as 3 years and anxiety disorders are as common in younger as older children (Egger & Angold, 2006).

Developmental factors, such as the ability to inhibit attention to threat (inhibition hypothesis; Kindt & Van Den Hout, 2001) and regulatory control (Salemink & Wiers, 2012) may moderate the association between negative interpretation and anxiety in adolescents and underpin age effects (see Field & Lester, 2010a; 2010bb for a more detailed discussion of potential moderating developmental factors). We were only able to investigate age as a proxy for development as, to date, there is a paucity of studies investigating the influence of specific developmental factors on the association between negative interpretation and anxiety. Another consideration is that findings may reflect age‐related differences in task *performance* rather than information processing per se (Field & Lester, 2010a). If younger children have difficulty understanding and completing the task as intended, this will likely lead to underestimated associations between negative interpretation and anxiety. In order for results from tasks to be reliable, the skills necessary for task completion must be sufficiently developed (Brown et al., 2013). Moving forward, it will be important for interpretation bias tasks to be designed in a developmentally sensitive way with studies ideally including assessments of relevant developmental factors alongside interpretation bias and anxiety.

### Evidence for content specificity

The finding that there was a larger association between bias and anxiety when anxiety subtype and scenario content matched than when they did not match provides evidence for content specificity in children and adolescents. Such evidence is in line with the cognitive specificity hypothesis (Beck, 1976) and adult reviews that have concluded that there is an association between emotions and mood‐congruent interpretation biases (Blanchette & Richards, 2009). Our results extend this finding to children and adolescents.

It is important to consider whether this finding relates to all anxiety disorders. Where studies had examined content specificity it was almost always for social anxiety with interpretation of social versus nonsocial scenarios. Therefore, it would be premature to suggest that this evidence for content specificity applies across anxiety disorders. Furthermore, this analysis is based upon primary anxiety diagnoses or anxiety symptoms and it is therefore unclear how the presence of comorbid anxiety disorders affect biases.

### Clinical implications

The moderate overall association between anxiety and negative interpretation confirms that it may be appropriate for anxiety treatments to include some focus on negative interpretation, at least in older children and adolescents. The finding that age significantly moderated the association between anxiety and negative interpretation suggests that, with age, the processing of ambiguity may become increasingly important as a focus within anxiety treatments and may be an important treatment target for adolescents with elevated anxiety. On the other hand, targeting negative interpretation may not be so central to the treatment of anxiety in younger children: for example, Thirlwall, Cooper, and Creswell (2017) found that for 7–12‐year‐olds undergoing parent‐guided cognitive‐behavioural therapy, child threat interpretation decreased from pre‐ to post‐treatment in both treated and wait list groups, and this change was not associated with recovery from primary anxiety diagnosis. It is possible that there are interactions between age and other moderating variables that would assist in elaborating on the clinical implications of the age effect. For example, age may interact with a match between scenario and anxiety type, whereby focusing on scenarios matching the child's anxiety in treatment may only be/be more appropriate for a particular age group. However, a lack of power in this study meant investigations of such interactions was not possible and would be an important consideration for future research.

The moderation by a match between scenario and anxiety subtype suggests targeting interpretations related to the child/adolescent's specific anxiety diagnosis may prove most efficacious. However, three things should be noted when considering the clinical implications of results. First, while the meta‐analysis did find a larger association between interpretation bias and anxiety when there was a match between scenario content and anxiety subtype, an association was still present when there was no match. This suggests that the targeting of interpretations, regardless of whether they do or do not reflect the anxiety subtype, may still be appropriate in treatment. Second, it is unclear whether age/development influences content specificity; it may be that targeting interpretations related to anxiety subtype may be more appropriate for some ages than others. Finally, the present findings are entirely based on cross‐sectional data and it is important to keep in mind that the *causal* relationship between negative interpretation and anxiety has not been confirmed by the present results. While experimental studies were included, effects sizes were only taken from associations at a single time point, as per the focus of this review. As such, the effect sizes included in this review are subject to the same issues that apply to correlational designs: unobserved confounding variables might account for the associations. Whether interpretation bias and anxiety are causally related and whether associations are unidirectional or reciprocal remains unclear. Hallion and Ruscio (2011) and Van Bockstaele et al.'s (2013) both found evidence to suggest a modest causal relationship between cognitive biases and anxiety, going from the bias to anxiety, among adults. Some studies with children and adolescents have also shown that successful manipulation of interpretation (using Cognitive Bias Modification of Interpretation; CBM‐I) is associated with changes in anxiety and fear (Lau, Belli, & Chopra, 2013; Lau, Pettit, & Creswell, 2013; Vassilopoulos, Banerjee, & Prantzalou, 2009), consistent with a causal pathway. However, a recent meta‐analysis concluded that changes in interpretation bias caused by CBM paradigms did not significantly affect symptoms of anxiety in children (Cristea, Mogoașe, David, & Cuijpers, 2015). Thus, there is scope for further work to examine the exact interplay between biases and anxiety and the conditions under which a causal association is found. The association between interpretation and attention biases is also unclear, with the majority of cognitive bias research focusing on one or other of these biases. It is possible that both biases share the same processing mechanism (Williams et al., 1997) or that one may directly influence the other (Hirsch, Clark, & Mathews, 2006), for example, attention bias may have a cascading influence on interpretation bias (Daleiden & Vasey, 1997; Muris & Field, 2008; White, Suway, Pine, Bar‐Haim, & Fox, 2011). Future research capturing the interaction between attention and interpretation bias in child anxiety over time would be beneficial. Furthermore, extending cognitive bias research to consider other biases such as confirmation bias may be a useful avenue for future research with a recent study suggesting a possible reciprocal relationship between bias and anxiety in children (Remmerswaal, Huijding, Bouwmeester, Brouwer, & Muris, 2014).

### Strengths and limitations

This meta‐analysis is the first to provide a systematic quantitative investigation of the size of the association between negative interpretation and anxiety in children and adolescents as well as the first to investigate whether particular variables influence this association. Quantitative investigations of publication bias suggest that results are unlikely to indicate a positive association where there is none. Also, 24 unpublished manuscripts/datasets were accepted from our request for unpublished work. The lack of significant moderation by publication status indicated that the effect size was robust, and not significantly different, across published and unpublished studies. We should acknowledge while we were reasonably successful in obtaining unpublished data, one can never be sure that the ‘file‐drawer’ has been completely emptied and thus can never rule out publication bias entirely. However, with the inclusion of 24 unpublished datasets and the presence of an effect within both published and unpublished data we feel reasonably confident the effect found here offers a fair reflection of the true relationship between interpretation bias and anxiety.

It should be noted that some planned moderation analyses for population variables could not be conducted. It was also not possible to assess moderation by inclusion or exclusion of those with comorbid depression because there were not enough effect sizes present within the excluded level (*k *=* *1). In addition, we chose not to conduct an analysis comparing state versus trait anxiety specifically because of the large discrepancy in numbers of effect sizes available for each level [state anxiety level (*k *=* *6), trait anxiety level (*k *=* *203)]. However, the moderation analysis by anxiety subtype included state anxiety and no significant difference in effect sizes was found across subtype. It is also important to note that the moderation by comorbid anxiety disorders may have been underpowered to detect a difference between the levels due to the small number of effect sizes within the ‘excluded’ level (*k *=* *5) (‘included’ level, *k *=* *77). However, inspection of the effect sizes shown in Table 2 support the lack of significant difference found by this moderation analysis.

The meta‐analysis was powered to investigate its main aims, but it is important to note that few studies and effect sizes were included in some analyses affecting the generalisability of findings and some moderation analyses may have been underpowered to find an effect. This is particularly relevant to the analysis of whether effect sizes vary across clinical disorders and across different control groups. Therefore, conclusions from these analyses may not be generalisable beyond this meta‐analysis and may warrant further investigation. The issue of power is also relevant to levels of certain moderators that contained a small number of studies. Specifically, the moderator levels of concern are identified by a superscript ‘c’ in Table 2. Should more effect sizes become available from new studies these moderation questions may be revisited.

To ensure a focused review, we did not examine the potential association between anxiety and positive interpretation or distress ratings. There is some evidence from adults, particularly within social anxiety, that the difference in interpretation bias between anxious and nonanxious individuals may be a *lack* of a positive bias (Gutiérrez‐García & Calvo, 2014; Moser, Huppert, Foa, & Simons, 2012). Among the studies accepted for this meta‐analysis, seven suggested that there was no association between anxiety and positive interpretations (Bögels & Zigterman, 2000; Dodd, 2012; Klein et al., 2014; Levin, 2008; Miers, Blöte, Bögels, & Westenberg, 2008; Schneider, In‐albon, Rose, & Ehrenreieh, 2006). However, in line with adult studies, three found nonanxious children rated positive outcomes as more likely than anxious children (De Hullu, 2012; Haller, Raeder, Scerif, Cohen Kodash, & Lau, 2016; Pile & Lau, 2015). Distress ratings, from ambiguous scenarios tasks, were not included among the dependent variables in this meta‐analysis because it did not conform to our operationalisation of negative interpretation. However, anticipated distress has been found to be associated with anxiety in children (Creswell & O'Connor, 2006; Marques, Pereira, Barros, & Muris, 2013; Vassilopoulos & Banerjee, 2012; Waters et al., 2008). Although beyond the scope of the present meta‐analysis, a thorough investigation of whether anxious children and adolescents show a lack of positive interpretation bias, and/or experience elevated anticipated distress when faced with ambiguous situations, may provide further insight into how anxious children and adolescents process ambiguity.

## Conclusion

This meta‐analysis provides the first quantitative systematic investigation of the association between negative interpretation and anxiety in children and adolescents. Results indicate a robust association between negative interpretation and anxiety in children and adolescents. Two moderators of this association were found: age and whether the scenario content matches the anxiety. The results expand age effects found in investigations of attention bias and anxiety in children and adolescents to another cognitive bias and broaden evidence of content specificity within this association from adults to children and adolescents. Future research and treatments should consider the impact of development on the relationship between interpretation bias and anxiety and whether evidence for content specificity holds across disorders.

**Key points**

- An association between interpretation bias and anxiety is consistently found in adults; however, evidence for this association in children and adolescents is inconsistent.
- This is the first systematic, quantitative investigation of the magnitude of the association between interpretation bias and anxiety in children and adolescents including moderators.
- Overall a robust medium positive association was found. Two influential moderators were found. Moderation by age suggested the association strengthens with age. Match between scenario content and anxiety subtype supported content specificity.
- Moderation results encourage consideration of the influence of development and content specificity on treatment in this population.
- The majority of studies included used a correlational design and there were few studies with young children.

## Supporting information


**Appendix S1**. Table of coding criteria and descriptions of each criterion.
**Appendix S2**. Search terms.
**Appendix S3**. References included within the meta‐analysis.

## Notes

1 After discussion between authors, any paper utilising the Children's Negative Cognitive Errors Questionnaire (Leitenberg, Yost, & Carroll‐Wilson, 1986) were excluded from the analysis given the interpretation of ambiguity could not be directly extracted from the measure.

2 There were no papers where coder 1 accepted and coder 2 rejected resulting in an empty cell so it was not possible to calculate a kappa value.
