# Supplementary material for: Research Review: Is anxiety associated with negative interpretations of ambiguity in children and adolescents? A systematic review and meta‐analysis
Source: J Child Psychol Psychiatry. 2017 Oct 20;59(11):1127–42. doi: 10.1111/jcpp.12822 (PMC6849625; doi:10.1111/jcpp.12822)
Supplement: Supplementary file 1 — Appendix S1. Table of coding criteria and descriptions of each criterion. Appendix S2. Search terms. Appendix S3. References included within the meta‐analysis. [file JCPP-59-1127-s001.docx]

Appendix S1

Table of Coding Criteria and Descriptions of each Criterion

| Table A1.  *Descriptions of Coding Criteria Applied to Obtain Appropriate Sample Characteristics and Information Regarding Moderator Variables.* | | |
| --- | --- | --- |
| Sample Characteristic | | Description of coding |
| Age | | age range, mean age, standard deviation of age were all coded to represent the sample’s age |
| Gender | | % of males in the sample |
| Ethnicity | | % of each identified ethnic group within the sample |
| Socio-economic status (SES) | | % within categories of SES |
|  | |  |
| Moderators and Level Names | | Description of coding within the level |
| Population Focus | |  |
|  | Clinical | The focus of the study was to assess interpretation bias in a clinically anxious population. |
|  | Community | The focus of the study was to assess interpretation bias within a normative sample. |
| Control Group | |  |
|  | Screened Non-Anxious control | Studies with a clinical group, where the comparison group consisted of children and adolescents screened to be non-anxious using a standardised measure of anxiety/fear. |
|  | Diagnosed Non-Anxious | Studies with a clinical group, where the comparison group consisted of children and adolescents with no clinical anxiety diagnosis as deemed by a clinical diagnostic measure. |
|  | Not *target anxiety disorder*, i.e. Not Social Anxiety, Not Separation Anxiety | Studies that compared participants with a specific anxiety disorder (X) to those with another anxiety disorder (not X). The comparison group was then coded ‘Not X anxiety’. |
|  | Clinical Externalising | Studies where the comparison group, for a group of clinically anxious children and adolescents consisted of children and adolescents with a diagnosed externalising disorder. |
|  | High Trait Anxiety | Studies with a clinical group, where the comparison group consisted of children and adolescents from the community screened as having high anxiety. |
|  | Low Trait Anxiety | Studies with a clinical group, where the comparison group consisted of children and adolescents from the community screened as having low anxiety. |
|  | Correlation | If an effect size was drawn from correlation analyses. |
| Comorbidity with Other Anxiety Disorder | |  |
|  | Included | Those with more than one diagnosed anxiety disorder were included in the clinically anxious group. |
|  | Excluded | Those with an anxiety disorder other than the disorder of interest or had more than one diagnosis where excluded from the clinically anxious group. |
| Comorbidity with Depression | |  |
|  | Included | Those with comorbid diagnosed clinical depression were included in the clinically anxious group. |
|  | Excluded | Those with comorbid diagnosed clinical depression were excluded from the clinically anxious group. |
| Comorbidity with Another Disorder | |  |
|  | Included | Those with a comorbid diagnosed psychiatric disorder (other than depression) were included from the clinically anxious group. |
|  | Excluded | Those with a comorbid diagnosed psychiatric disorder (other than depression) were excluded from the clinically anxious group. |
| Anxiety Subtype | |  |
|  | General Anxiety | Studies where no specific subtype was assessed, including those that assessed general trait anxiety as the concept of interest. |
|  | OCD | Studies where the target group had high symptoms or a diagnosis of obsessive compulsive disorder. |
|  | Phobias | Studies where the target group had high symptoms or a diagnosis of a specific phobia. |
|  | Separation Anxiety | Studies where the target group had high symptoms or a diagnosis of separation anxiety. |
|  | Social Anxiety | Studies where the target group had high symptoms or a diagnosis of social anxiety. |
|  | State Anxiety | Studies where the target group was assessed for levels of state of anxiety. |
|  | Other Anxiety | Studies where the anxious group was defined by the absence of a specific anxiety disorder (e.g. Social Anxiety Disorder), but had (symptoms of) another anxiety disorder. |
|  | PTSD | Studies where the target group had high symptoms or a diagnosis of post traumatic stress disorder. |
| Task Type | |  |
|  | Ambiguous Scenarios | The task used to assess interpretation bias involved responding to a set of ambiguous scenarios e.g. the ASQ. |
|  | Lexical Tasks | The task used to assess interpretation bias involved responding to language based stimuli e.g. homophone/graph task, lexical decision task. |
| Response Type**^b^** | |  |
|  | Forced Choice | Participants responded to the interpretation bias task by selecting a response from a provided set of responses that included a threat/negative interpretation. |
|  | Open | Participants responded to the interpretation bias task by providing an open response (usually their interpretation in their own words), which was then coded, for example, as a threat/negative interpretation. |
|  | Open and Forced Choice | Scores calculated from open and forced choice responses were combined to create a composite score of threat/negative interpretation. |
| Dependent Variable^a^ | |  |
|  | Threat Interpretation | Responses to ambiguous scenarios are coded as reflecting a negative or threat interpretation via forced choice or open question. |
|  | Threat Threshold | The number of sentences taken to describe an ambiguous scenario before the child or adolescent stated the story was scary/threatening/negative. |
|  | Threat Frequency | Out of all the sentences of the story read to the child/adolescent: the total number of sentences after which the child/adolescent identified the scenario as threatening. |
| Scenario Type^a^ | |  |
|  | Social | The scenarios were described as have a social element or as being relevant to social anxiety and a given example scenario confirmed this description. |
|  | General | The scenarios were described as non-social, without physical information (see below), reflect generalised anxiety, or total scenario scores from studies that have not reported separate effects from scenario subtypes. |
|  | Separation | The scenarios were described as involving some form of separation from another person or being relevant to separation anxiety and a given example scenario confirmed this description. |
|  | Phobias | The scenarios were described as providing information that could be resolved by assuming the presence of a feared object or as being relevant to panic disorder and a given example scenario confirmed this description. |
|  | Physical Information | The scenarios were described as involving reference to physical sensations of the participant i.e. heart beating, poorly stomach and a given example scenario confirmed this description. |
| Match: Scenario and Anxiety Subtype^a^ | |  |
|  | No Match | Given the description and examples of scenarios used in the study the content of the ambiguous scenario did not directly reflect the anxiety subtype under investigation. |
|  | Match | Given the description and examples of scenarios used in the study the content of the ambiguous scenario directly reflected the anxiety subtype under investigation. |
| Anxiety Measure Informant | |  |
|  | Child | The child or adolescent responded to the anxiety/fear questionnaire measure or was interviewed to assess clinical anxiety. |
|  | Parent | The child or adolescent’s parent responded to the anxiety/fear questionnaire measure or was interviewed to assess clinical anxiety. |
|  | Child and Parent | The results from both child/adolescent and parent clinical anxiety interviews or anxiety/fear questionnaires were combined to create a composite. |
|  | Teacher | The child or adolescent’s teacher responded to anxiety/fear questionnaire measure or was interviewed to assess clinical anxiety. |
| *Note.* ^a^ These criteria only applied to studies utilising an ambiguous scenarios tasks to assess interpretation bias. ^b^ Responses were only included regarding a negative or threat interpretation. While it is acknowledged the positive interpretations and distress ratings are possible responses to ambiguous scenarios and lexical tasks they are beyond the scope of this review. | | |

Appendix S2

Search Terms

1. Search terms to identify those related to interpretation bias and anxiety:

(Interpret* bias OR Interpretation of ambig* OR bias* interpret* OR interpret ADJ bias* OR “Reduced evidence for danger” OR “Threat perception bias”) AND (anxi* OR worry OR fear OR obses* OR compul* OR OCD OR panic OR anxi* disorder OR GAD OR generali* anxiety disorder OR phobi*). The other set was: (cognitive bias modification AND interpret*) AND (anxi* OR worry OR fear OR obses* OR compul* OR OCD OR panic OR anxi* disorder OR GAD OR generali* anxiety disorder OR phobi*)

1. Search terms to identify those specifically related to cognitive bias modification:

(cognitive bias modification AND interpret*) AND (anxi* OR worry OR fear OR obses* OR compul* OR OCD OR panic OR anxi* disorder OR GAD OR generali* anxiety disorder OR phobi*)

Appendix S3

References included within the Meta-analysis

*Indicates that the data or paper was provided by the authors under the requested for unpublished data and was unpublished at the time the meta-analysis was conducted.

Alkozei, A., Cooper, P. J., & Creswell, C. (2014). Emotional reasoning and anxiety sensitivity: Associations with social anxiety disorder in childhood. *Journal of Affective Disorders*, *152-154*, 219–228. doi:10.1016/j.jad.2013.09.014

Bögels, S. M., van Dongen, L., & Muris, P. (2003). Family influences on dysfunctional thinking in anxious children. *Infant and Child Development*, *12*(3), 243–252. doi:10.1002/icd.288

Bögels, S. M., & Zigterman, D. (2000). Dysfunctional cognitions in children with social phobia, separation anxiety disorder, and generalized anxiety disorder*.* *Journal of Abnormal Child Psychology* *28*(2), 205-211. doi:10.1023/A:1005179032470

Carthy, T., Horesh, N., Apter, A., & Gross, J. J. (2010). Patterns of emotional reactivity and regulation in children with anxiety disorders. *Journal of Psychopathology and Behavioral Assessment*, *32*(1), 23–36. doi:10.1007/s10862-009-9167-8

Cederlund, R., & Ost, L. G. (2011). Perception of threat in children with social phobia: comparison to nonsocially anxious children before and after treatment. *Journal of Clinical Child and Adolescent Psychology,* *40*(6), 855–63. doi:10.1080/15374416.2011.618448

Chan, S. W. Y., Lau, J. Y. F., & Reynolds, S. A. (2014). Is cognitive bias modification training truly beneficial for adolescents? *Journal of Child Psychology and Psychiatry*, *56*(11), 1239-1248. doi:10.1111/jcpp.1236

Chorpita, B. F., Albano, A. M., & Barlow, D. H. (1996). Cognitive processing in children: Relation to anxiety and family influences. *Journal of Clinical Child Psychology*, *25*(2), 170–176. doi: http://dx.doi.org/10.1207/s15374424jccp2502_5

*Cox, P., Bamford, G. M., & Lau, J. Y. F. (2015). Cognitive bias modification as a strategy to reduce children’s fears and concerns about the secondary school transition. *Anxiety, Stress, & Coping*, *29*(4) , 447-456. doi:10.1080/10615806.2015.1058367

Creswell, C., Murray, L., & Cooper, P. (2014). Interpretation and expectation in childhood anxiety disorders: Age effects and social specificity. *Journal of Abnormal Child Psychology*, *42*(3), 453–65. doi:10.1007/s10802-013-9795-z

Creswell, C., & O’Connor, T. G. (2006). “Anxious cognitions” in children: An exploration of associations and mediators. *British Journal of Developmental Psychology*, *24*(4), 761–766. doi:10.1348/026151005X70418

Creswell, C., & O’Connor, T. G. (2011). Interpretation bias and anxiety in childhood: Stability, specificity and longitudinal associations. *Behavioural and Cognitive Psychotherapy*, *39*(2), 191–204. doi:10.1017/S1352465810000494

Creswell, C., Schniering, C. A, & Rapee, R. M. (2005). Threat interpretation in anxious children and their mothers: Comparison with nonclinical children and the effects of treatment. *Behaviour Research and Therapy*, *43*(10), 1375–81. doi:10.1016/j.brat.2004.10.009

Creswell, C., Shildrick, S., & Field, A. P. (2011). Interpretation of ambiguity in children: A prospective study of associations with anxiety and parental interpretations. *Journal of Child and Family Studies*, *20*(2), 240–250. doi:10.1007/s10826-010-9390-7

*Dobrean, A. (2015). *ASQ and SCARED.* Unpublished raw data.

*Dodd, H.F. (2012). *Interpretation bias and anxiety in adolescents****.*** Unpublished raw data.

Eley, T. C., Gregory, A. M., Lau, J. Y. F., McGuffin, P., Napolitano, M., Rijsdijk, F. V, & Clark, D. M. (2008). In the face of uncertainty: a twin study of ambiguous information, anxiety and depression in children. *Journal of Abnormal Child Psychology*, *36*(1), 55–65. doi:10.1007/s10802-007-9159-7

Field, Z. C., & Field, A. (2013). How trait anxiety, interpretation bias and memory affect acquired fear in children learning about new animals. *Emotion*, *13*(3), 409–423. doi:10.1037/a0031147

*Fu, X., Du, Y., Au, S., & Lau, J. Y. F. (2015). Single-session cognitive bias modification of interpretations training in high-anxious adolescents. *Journal of Cognitive Psychology*, *29*(3), 253–272.

Gifford, S., Reynolds, S., Bell, S., & Wilson, C. (2008). Threat interpretation bias in anxious children and their mothers. *Cognition & Emotion*, *22*(3), 497–508. doi:10.1080/02699930801886649

Haller, S. W. P., Raeder, S. M., Scerif, G., Cohen Kodash, K., & Lau, J. Y. F. (2016). Measuring online interpretations and attributions of social situations: links with adolescent social anxiety. *Journal of Behavior Therapy and Experimental Psychiatry*, *50*, 250–256. doi:http://dx.doi.org/10.1016/j.jbtep.2015.09.009

Hudson, J.L., & Dodd, H.F. (2010). *Interpretation bias and anxiety in middle childhood*. Unpublished raw data.

Hullu, E. D. (2012). Biased Interpretations of Ambiguous Social Situations in Socially Anxious Adolescents. In *Threat is in the eye of the beholder: cognitive bias modification in the prevention of adolescent social anxiety* (pp 43-50). Groningen: s.n.

In-Albon, T., Klein, A., Rinck, M., Becker, E., & Schneider, S. (2008). Development and evaluation of a new paradigm for the assessment of anxiety-disorder-specific interpretation bias using picture stimuli. *Cognition & Emotion*, *22*(3), 422–436. doi:10.1080/02699930701872293

In-Albon, T., Dubi, K., Rapee, R. M., & Schneider, S. (2009). Forced choice reaction time paradigm in children with separation anxiety disorder, social phobia, and nonanxious controls. *Behaviour Research and Therapy*, *47*(12), 1058–65. doi:10.1016/j.brat.2009.08.003

*In-Albon, T., Pfeiffer, S. & Schneider, S. (2016). *Interpretation bias in children with separation anxiety disorder: Psychometric properties of a disorder-specific questionnaire and effects of CBT*. Manuscript submitted for publication.

*Klein, A.M., de Voogd, E.L., Wiers, R.W., & Salemink, E. (2017). Biases in attention and interpretation in adolescents with varying levels of anxiety and depression*.* *Cognition and Emotion,* 1-9. DOI: 10.1080/02699931.2017.1304359

*Klein, A. M., Rapee, R. M., Hudson, J. F., Morris, T. M., Schneider, S. C., Schniering, C. A., Becker, E. S., & Rinck, M. (2014). *Content-specific interpretation biases in clinically anxious children.* Manuscript under review.

*Klein, A. M., Bakens, R., van Niekerk, R., Ouwens, M. A., Rapee, R. M., Bögels, S. M., Becker, E. S., & Rinck, M. (2014). *Children with symptoms of generalized anxiety disorder show a content-specific interpretation bias.* Manuscript under review.

Klein, A. M., Titulaer, G., Simons, C., Allart, E., de Gier, E., Bögels, S. M., … Rinck, M. (2014). Biased interpretation and memory in children with varying levels of spider fear. *Cognition & Emotion*, *28*(1), 182–92. doi:10.1080/02699931.2013.810144

Lau, J. Y. F., Pettit, E., & Creswell, C. (2013). Reducing children’s social anxiety symptoms: Exploring a novel parent-administered cognitive bias modification training intervention. *Behaviour Research and Therapy*, *51*(7), 333–337. doi:10.1016/j.brat.2013.03.008

Lester, K. J., Seal, K., Nightingale, Z. C., & Field, A. P. (2010). Are children’s own interpretations of ambiguous situations based on how they perceive their mothers have interpreted ambiguous situations for them in the past? *Journal of Anxiety Disorders*, *24*(1), 102–8. doi:10.1016/j.janxdis.2009.09.004

Levin, L. (2008). *Mechanisms linking early behavioral inhibition to later social functioning : The role of autobiographical memory biases*. University of Miami, Florida.

Loscalzo, Y., & Giannini, M. (2015). Social anxiety disorder prevention in adolescence. Psychometric properties of the adolescents’ interpretation and belief questionnaire (AIBQ). *Counseling*, *8*(2). Retrieved from http://rivistedigitali.erickson.it/counseling/en/archivio/vol-8-n-2/

Lu, W., Daleiden, E., Pratt, S., Shay, A., Stone, B., & Asaku-Yeboah, M. (2013). Life events and internalizing problems among Chinese school children: An examination of the cognitive diathesis model. *Asian Journal of Social Psychology*, *16*(4), 307–319. doi:10.1111/ajsp.12036

Micco, J. A., & Ehrenreich, J. T. (2008). Children’s interpretation and avoidant response biases in response to non-salient and salient situations: relationships with mothers' threat perception and coping expectations. *Journal of Anxiety Disorders*, *22*(3), 371–385. doi:10.1016/j.janxdis.2007.03.009

Micco, J. A., Hirshfeld-Becker, D. R., Henin, A., & Ehrenreich-May, J. (2013). Content specificity of threat interpretation in anxious and non-clinical children. *Cognitive Therapy and Research*, *37*(1), 78–88. doi:10.1007/s10608-012-9438-7

*Micco, J. A. (2012). *Cognitive Biases in Offspring at Risk for Anxiety Disorders*. Unpublished raw data.

*Miers, A. (2014). *AIBQ and SAS-A 2014.* Unpublished raw data.

Miers, A. C., Blöte, A. W., Bögels, S. M., & Westenberg, P. M. (2008). Interpretation bias and social anxiety in adolescents. *Journal of Anxiety Disorders*, *22*(8), 1462–71. doi:10.1016/j.janxdis.2008.02.010

Mogoase, C., Podina, I. R., Sucala, M., & Dobrean, A. (2013). Evaluating the unique contribution of irrational beliefs and negative bias. *PsycINFOJournal of Cognitive and Behavioral Psychotherapies*, *13*(2), 465–475. Retrieved from http://search.proquest.com.idpproxy.reading.ac.uk/docview/1470064313?accountid=13460

Muris, P., Huijding, J., Mayer, B., Remmerswaal, D., & Vreden, S. (2009). Ground control to Major Tom: experimental manipulation of anxiety-related interpretation bias by means of the “space odyssey” paradigm and effects on avoidance tendencies in children. *Journal of Anxiety Disorders*, *23*(3), 333–40. doi:10.1016/j.janxdis.2009.01.004

Muris, P., Jacques, P., & Mayer, B. (2004). The stability of threat perception abnormalities and anxiety disorder symptoms in non-clinical children. *Child Psychiatry and Human Development*, *34*(3), 251–265. doi:10.1023/B:CHUD.0000015000.13845.9d

Muris, P., Kindt, M., Bögels, S., Merckelbach, H., Gadet, B., & Moulaert, V. (2000). Anxiety and threat perception abnormalities in normal children. *Journal of Psychopathology and Behavioral Assessment*, *22*(2), 183–199. doi:10.1023/A:1007588524525

Muris, P., Luermans, J., & Merckelbach, H. (2000). ‘Danger is lurking everywhere’. The relation between anxiety and threat perception abnormalities in normal children. *Journal of Behavior Therapy and Experimental Psychiatry*, *31,* 123-136. doi: http://dx.doi.org/10.1016/S0005-7916(00)00016-1

Muris, P., Mayer, B., van Eijk, S., & van Dongen, M. (2007). “I’m not really afraid of Osama Bin Laden!” fear of terrorism in Dutch children. *Journal of Child and Family Studies*, *17*(5), 706–713. doi:10.1007/s10826-007-9185-7

Muris, P., Meesters, C., Smulders, L., & Mayer, B. (2005). Threat perception distortions and psychopathological symptoms in typically developing children. *Infant and Child Development*, *14*(3), 273–285. doi:10.1002/icd.392

Muris, P., Merckelbach, H., & Damsma, E. (2000). Threat perception of nonreferred, socially anxious children. *Journal of Clinical Child Psychology*, *29*(3), 348–359. doi:10.1207/s15374424jccp2903_6

Muris, P., Merckelbach, H., Schepers, S., & Meesters, C. (2003). Anxiety, threat perception abnormalies and emotional reasoning in nonclinical dutch children. *Journal of Clinical Child & Adolescent Psychology*, *32*(3), 453–459.

Muris, P., Rapee, R., Meesters, C., Schouten, E., & Geers, M. (2003). Threat perception abnormalities in children: The role of anxiety disorders symptoms, chronic anxiety, and state anxiety. *Journal of Anxiety Disorders*, *17*(3), 271–287. doi:http://dx.doi.org/10.1016/S0887-6185(02)00199-8

Muris, P., & van Doorn, M. (2003). “Danger is lurking everywhere, even in parts of a jigsaw puzzle”: Anxiety-related threat perception abnormalities in children: Their assessment with projective material. *Behaviour Change*, *20*(3), 151–159. doi:10.1375/bech.20.3.151.24835

Ooi, J., Dodd, H. F., & Walsh, J. (2015). Shared cognition in childhood anxiety: interpretation bias in preschool children and their parents. *Journal of Child and Family Studies*. doi:10.1007/s10826-015-0143-5

*Ooi, L. (2012). *Exploring links between shyness, interpretation biases, and negative peer experiences in early childhood*.

*Păsăreu, C., Podina, I., Mogoașe, C., Balazsi, R., Dobrean, A. (2015). *The intergenerational transmission of worry: the mediating role of child’s interpretation biases.* Manuscript under review.

*Pearcey, S. (2014). *An examination of interpretation bias in anxious children: Overcoming limitations with a memory recall task*. University of Reading.

Pereira, A. I., Barros, L., Mendonça, D., & Muris, P. (2014). The relationships among parental anxiety, parenting, and children’s anxiety: the mediating effects of children's cognitive vulnerabilities. *Journal of Child and Family Studies*, *23*(2), 399–409. doi:10.1007/s10826-013-9767-5

*Pereira, A.I., Marques, T, Barros, L. (2016) *Cognitive biases and coping strategies in anxious and non-anxious school aged children*. Manuscript submitted for publication.

*Pile, V. & Lau, J. Y. F (2015). *Interpretation Biases and Social Anxiety in Adolescence.* Unpublished raw data.

Podină, I. (2013). Intergenerational transmission of anxiety: evidence for the mediating role of the negative interpretation bias. *Journal of Cognitive and Behavioural Psychotherapies*, *13*(2), 309–320. Retrieved from http://www.researchgate.net/publication/257030425_Intergenerational_transmission_of_anxiety_evidence_for_the_mediating_role_of_the_negative_interpretation_bias/file/60b7d5283631f6bc1b.pdf

Reid, S. C., Salmon, K., & Lovibond, P. F. (2006). Cognitive biases in childhood anxiety, depression, and aggression: are they pervasive or specific? *Cognitive Therapy and Research*, *30*(5), 531–549. doi:10.1007/s10608-006-9077-y

Salemink, E., & Wiers, R. W. (2011). Modifying threat-related interpretive bias in adolescents. *Journal of Abnormal Child Psychology*, *39*(7), 967–976. doi:10.1007/s10802-011-9523-5

Salemink, E., & Wiers, R. W. (2012). Adolescent threat-related interpretive bias and its modification: the moderating role of regulatory control. *Behaviour Research and Therapy*, *50*(1), 40–6. doi:10.1016/j.brat.2011.10.006

Schneider, S., In-albon, T., Rose, U., & Ehrenreieh, J. T. (2006). Measurement of panic interpretation bias using the anxiety interpretation questionnaire for children. *Journal of Cognitive Psychotherapy*, *20*(1), 85–96.

Shortt, A. L., Barrett, P. M., Dadds, M. R., & Fox, T. L. (2001). The influence of family and experimental context on cognition in anxious children. *Journal of Abnormal Child Psychology*, *29*(6), 585–596. doi:10.1023/A:1012289427845

Smári, J., Pétursdóttir, G., & Porsteinsdóttir, V. (2001). Social anxiety and depression in adolescents in relation to perceived competence and situational appraisal. *Journal of Adolescence*, *24*(2), 199–207. doi:10.1006/jado.2000.0338

Smith-Janik, S. B., Green, J.S., & Teachman, B. A. (2013). Age Differences in Information Processing Biases in Spider Fear. *International Journal of Cognitive Therapy 6*(4), 401-420. doi: http://dx.doi.org/10.1521/ijct.2013.6.4.401

Suarez-Morales, L., & Bell, D. (2006). Relation of childhood worry to information-processing factors in an ethnically diverse community sample. *Journal of Clinical Child & Adolescent Psychology*, *35*(1), 136–147. doi:10.1207/s15374424jccp3501_12

Taghavi, M. R., Moradi, A. R., Neshat-Doost, H. T., Yule, W., & Dalgleish, T. (2000). Interpretation of ambiguous emotional information in clinically anxious children and adolescents. *Cognition and Emotion*, *14*(6), 809–822. doi:10.1080/02699930050156645

Varela, R. E., Vernberg, E. M., Sanchez-sosa, J. J., Riveros, A., & Mashunkashey, J. (2004). Anxiety reporting and culturally associated interpretation biases and cognitive schemas : A comparison of Mexican , Mexican American, and European American families. *Journal of Clinical Child & Adolescent Psychology Anxiety*, *2*(33), 237–247. doi:10.1207/s15374424jccp3302

Vassilopoulos, S. P., & Banerjee, R. (2012). Social anxiety and content specificity of interpretation and judgemental bias in children. *Infant and Child Development*, *21*(3), 298–309. doi:10.1002/icd.746

Vassilopoulos, S. P., Blackwell, S. E., Moberly, N. J., & Karahaliou, E. (2012). Comparing imagery and verbal instructions for the experimental modification of interpretation and judgmental bias in children. *Journal of Behavior Therapy and Experimental Psychiatry*, *43*(1), 594–601. doi:10.1016/j.jbtep.2011.08.004

*Vassilopoulos, S. P., & Brouzos, A. (2015). Cognitive bias modification of interpretations in children: Processing information about ambiguous social events in a duo. *Journal of Child and Family Studies*, *25*(1), 299- 307 doi: http://dx.doi.org/10.1007/s10826-015-0194-7

*Vassilopoulos, S. P., Moberly, N. J., & Lau, J. Y. F. (2015). Cognitive bias modification training in children affects anxiety during anticipatory processing of social evaluation. *International Journal of Cognitive Therapy*, *8*(4), 318–334. doi:10.1521/ijct.2015.8.4.318

Waite, P., Codd, J., & Creswell, C. (2015). Interpretation of ambiguity: Differences between children and adolescents with and without an anxiety disorder. *Journal of Affective Disorders*, *188*, 194–201. doi:http://dx.doi.org/10.1016/j.jad.2015.08.022

Waters, A. M., Wharton, T. a, Zimmer-Gembeck, M. J., & Craske, M. G. (2008). Threat-based cognitive biases in anxious children: comparison with non-anxious children before and after cognitive behavioural treatment. *Behaviour Research and Therapy*, *46*(3), 358–74. doi:10.1016/j.brat.2008.01.002

Waters, A. M., Zimmer-Gembeck, M. J., & Farrell, L. J. (2012). The relationships of child and parent factors with children’s anxiety symptoms: parental anxious rearing as a mediator. *Journal of Anxiety Disorders*, *26*(7), 737–45. doi:10.1016/j.janxdis.2012.06.002
